# A six-year study in a real-world population reveals an increased incidence of dyslipidemia during COVID-19

**DOI:** 10.1172/JCI183777

**Published:** 2024-09-12

**Authors:** Valentina Trimarco, Raffaele Izzo, Stanislovas S. Jankauskas, Mario Fordellone, Giuseppe Signoriello, Maria Virginia Manzi, Maria Lembo, Paola Gallo, Giovanni Esposito, Roberto Piccinocchi, Francesco Rozza, Carmine Morisco, Pasquale Mone, Gaetano Piccinocchi, Fahimeh Varzideh, Bruno Trimarco, Gaetano Santulli

**Affiliations:** 1 Department of Neuroscience, Reproductive Sciences, and Dentistry and; 2 Department of Advanced Biomedical Sciences, “Federico II” University, Naples, Italy.; 3 Department of Molecular Pharmacology, Fleischer Institute for Diabetes and Metabolism (FIDAM), Einstein Institute for Aging Research, Albert Einstein College of Medicine, New York, New York, USA.; 4 Department of Mental, Physical Health and Preventive Medicine and; 5 Department of Experimental Medicine, University of Campania “Luigi Vanvitelli,” Naples, Italy.; 6 “Luigi Vanvitelli” Hospital, Naples, Italy.; 7 Italian Society for Cardiovascular Prevention (SIPREC), Rome, Italy.; 8 International Translational Research and Medical Education (ITME), Academic Research Unit, Naples, Italy.; 9 Department of Medicine and Health Sciences “Vincenzo Tiberio,” Molise University, Campobasso, Italy.; 10 Casa di Cura Montevergine, Mercogliano, Avellino, Italy.; 11 COMEGEN Primary Care Physicians Cooperative, Italian Society of General Medicine (SIMG), Naples, Italy.; 12 Department of Medicine, Division of Cardiology, Einstein–Mount Sinai Diabetes Research Center (ES-DRC), Wilf Family Cardiovascular Research Institute, Albert Einstein College of Medicine, New York, New York, USA.

**Keywords:** Cardiology, Metabolism, Atherosclerosis, Cholesterol, Clinical practice

## Abstract

**BACKGROUND:**

Recent studies conducted in individuals who survived COVID-19 suggest that SARS-CoV-2 infection is associated with an increased risk of dyslipidemia. However, it remains unclear whether this augmented risk is confirmed in the general population and how this phenomenon is affecting the overall burden of cardiometabolic diseases.

**METHODS:**

To address these aspects, we conducted a 6-year longitudinal study to examine the broader effects of COVID-19 on dyslipidemia incidence in a real-world population (228,266 individuals) residing in Naples in southern Italy. The pre–COVID-19 and COVID-19 groups were balanced for demographic and clinical factors using propensity score matching.

**RESULTS:**

Our analysis spans a period of 3 years during the COVID-19 pandemic (2020–2022), comparing dyslipidemia incidence with pre-pandemic data (2017–2019), with a follow-up of at least 1,095 days corresponding to 21,349,215 person-years. During the COVID-19 period, we detected an increased risk of developing any dyslipidemia when compared with the pre–COVID-19 triennium (OR = 1.29; 95% CI, 1.19–1.39). Importantly, these estimates were adjusted for comorbidities by a multivariate analysis.

**CONCLUSIONS:**

Taken together, our data reveal a notable rise in dyslipidemia incidence during the COVID-19 pandemic, suggesting the utility of establishing specialized clinical monitoring protocols for patients who survive COVID-19 to mitigate the risk of developing dyslipidemia.

## Abstract 2

A 6-year study in a large real-world population demonstrated a significant rise in dyslipidemia risk during the COVID-19 pandemic, emphasizing the need for monitoring survivors.

## Introduction

Recent reports suggest that SARS-CoV-2 infection could be associated with an increased risk of dyslipidemia (1–5). A large observational study conducted by FAIR Health in people with COVID-19, with no control group, reported that approximately 3% developed dyslipidemia after the first 30 days of infection (2). Similarly, Xu et al. (1) used the national health care databases of the US Department of Veterans Affairs to build (a) a cohort of participants who had a positive COVID-19 test and survived the first 30 days of infection between March 2020 and January 2021, (b) a noninfected contemporary control group that included individuals enrolled between March 2020 and January 2021, and (c) a historical control group with individuals enrolled between March 2018 and January 2019 (1). Compared with the noninfected contemporary control group, those in the COVID-19 cohort had higher risks and burdens of dyslipidemia in the post-acute phase of the SARS-CoV-2 infection. However, these data do not clarify whether and to what extent such an increased risk could influence the global burden of cardiometabolic disease and how it may affect health systems and health care costs. In fact, the data currently available in the literature merely refer to comparisons between groups of patients who had a clinically confirmed positive SARS-CoV-2 test versus individuals with similar demographic characteristics who did not have COVID-19 (6), thus assessing the effect of the long-term individual COVID-19 infection (post-acute sequelae) on the incidence of dyslipidemia. Yet, it should be emphasized that the attenuation of the severity of COVID-19 symptoms and the use of less sensitive self-tests have made the less reliable evaluation of this complex phenomenon and its consequences for public health organizations in different countries (7).

Therefore, the present study was designed to examine the broader effects of the pandemic on dyslipidemia incidence in a real-world population residing in Naples, southern Italy. This analysis spans a period of 3 years during the pandemic (2020–2022), tracking individuals under the care of primary care physicians (8). We juxtaposed these findings with data from a pre–COVID-19 era population obtained from the same database, covering the period from 2017 to 2019.

## Results

In this longitudinal cohort study, we harnessed data obtained from COMEGEN (COoperativa di MEdicina GENerale: General Medicine Cooperative), an association of primary physicians in the Naples Local Health Authority of the Italian Ministry of Health (ASL Napoli 1 Centro) (9). Founded in 1997, COMEGEN today comprises 140 physicians who are linked in a network by using the same software, building a database that holds medical records of more than 200,000 adults (9). The territorial distribution of the individuals assisted by these physicians is similar to that of the city population recorded by the Italian Institute of Statistics, with no major differences in terms of aggregation by age or geographic area (10). We collected data from January 1, 2017 to December 31, 2022 (see further details in Methods). Anonymized data from electronic records of 228,266 patients in the 2017–2022 period were considered for the study and included 31,764 individuals with a confirmed diagnosis of COVID-19. Dyslipidemia outcomes consisted of either incident abnormal lipid laboratory results (i.e., total cholesterol above 200 mg/dL, HDL cholesterol below 40 mg/dL, or triglycerides above 150 mg/ dL) or incident lipid-lowering medication prescriptions (i.e., prescription of fibrates and/or statins). After excluding individuals with less than 3 observation/year in each of the 2 study periods (2017–2019 and 2020–2022) and those satisfying our criteria for dyslipidemia on January 1, 2017 and January 1, 2020, we obtained data on a cohort of 26,366 participants between 2017 and 2022 encompassing 2,201 individuals diagnosed with COVID-19.

**Figure 1:**
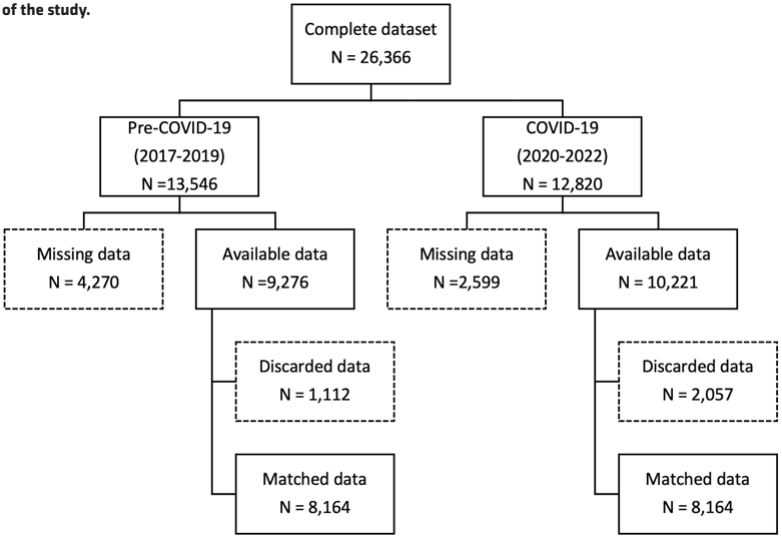
Flow chart of the study.

The pre–COVID-19 group included 13,546 participants enrolled between January 1, 2017 and December 1, 2019, whereas the COVID-19 time group included 12,820 individuals enrolled between January 1, 2020 and December 31, 2022. A total of 4,270 and 2,599 individuals were excluded from the 2 groups, respectively, due to missing data, so that we had data available for 9,276 individuals in the pre–COVID-19 triennium and 10,221 in the COVID-19 period (Figure 1), with a follow-up time of at least 1,095 days corresponding to 21,349,215 person-years. No difference in the frequency of lipid testing was detected in the 2 study periods. The demographic and clinical characteristics are presented in Table 1. As expected, the pre–COVID-19 population was younger; in addition, it had a larger percentage of smokers and a lower value of mean systolic and diastolic blood pressure (BP).

**Figure 2:**
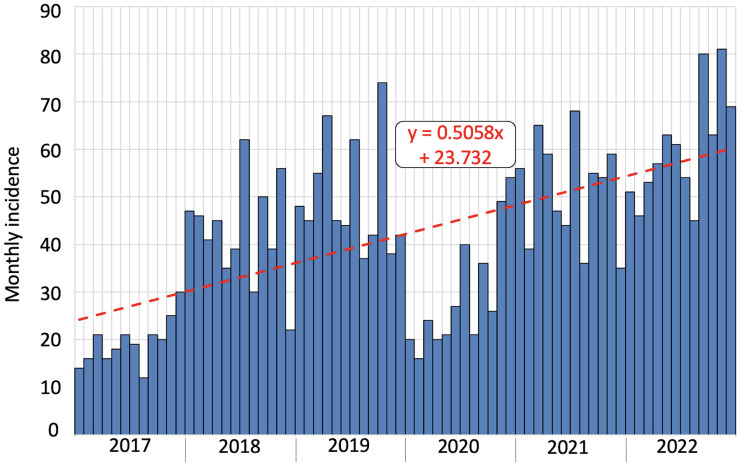
Monthly incidence of dyslipidemia in our population during the 6-year observation period. Each bar indicates 1 month.

Figure 2 illustrates the monthly dyslipidemia incidence for the complete 6-year follow-up period; a regression of the observed data against the counterfactual line was performed. The coefficients estimated with the linear regression model revealed a significant effect of time on the incidence of dyslipidemia, with a significant change in its slope (coefficient *b* = 0.506, *P* = 0.01).

The pre–COVID-19 and COVID-19 groups were balanced using the propensity score method. The clinical and demographic characteristics of these groups after weighting are shown in Table 2. Evaluation of standardized mean differences of these characteristics after weighting revealed differences equal to zero, with a CI of 95% (i.e., all the CIs included the zero value), suggesting good balance.

**Figure 3:**
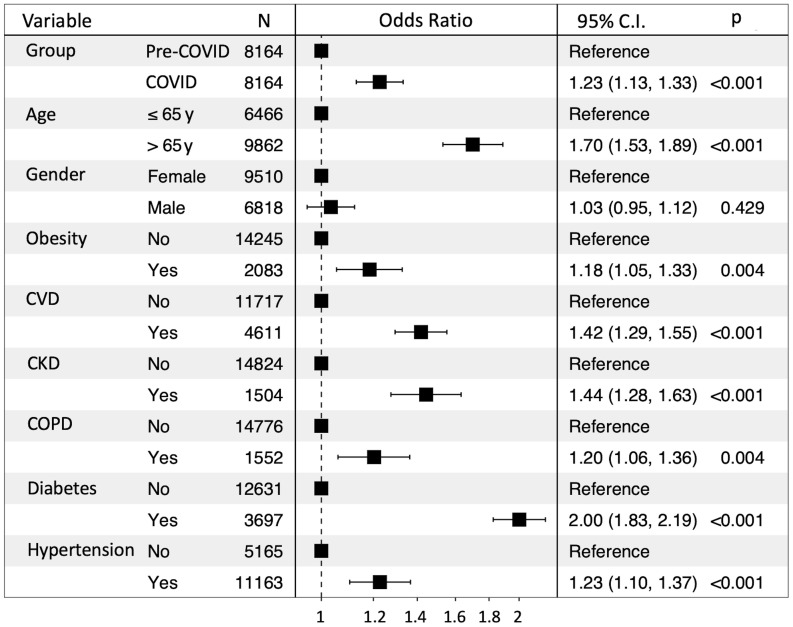
ORs for dyslipidemia in the pre–COVID-19 and COVID-19 cohorts adjusted for demographic factors and comorbidities.

We then compared the risks of prespecified dyslipidemia outcomes between the COVID-19 and pre–COVID-19 groups. Among the total 3,038 patients with dyslipidemia, 1,694 belonged to the COVID-19 cohort, while 1,344 were in the pre–COVID-19 cohort. The risks associated with dyslipidemia outcome are illustrated in Figure 3. Compared with the pre–COVID-19 triennium, we detected a significantly increased risk of patients developing any dyslipidemia outcome during the COVID-19 period (OR = 1.29, 95% CI 1.19–1.39).

Table 2 also shows the prevalence of comorbidities in the overall population in the pre–COVID-19 triennium and during the COVID-19 pandemic. In the latter period, there was an increased prevalence of all the considered pathologies, which achieved statistical significance except for chronic obstructive pulmonary disease (COPD). Hence, in order to rule out the possibility that the increased risk of dyslipidemia could be a mere clustering of cardiometabolic risk augmentation as opposed to an independent phenomenon, we adjusted this parameter for demographic factors as well as for comorbidities by a multivariate analysis. Specifically, the increased risk of an incident composite dyslipidemia outcome was evident in subgroups based on age class (OR = 1.54, 95% CI 1.40–1.70), obesity (OR = 1.22, 95% CI 1.09–1.36), cardiovascular disease (CVD) (OR = 1.29, 95% CI 1.18–1.40), chronic kidney disease (CKD) (OR = 1.41, 95% CI 1.25–1.59), COPD (OR = 1.20, 95% CI 1.07–1.36), diabetes (OR = 2.07, 95% CI 1.90–2.25), and hypertension (OR = 1.30, 95% CI 1.18–1.43).

**Figure 4:**
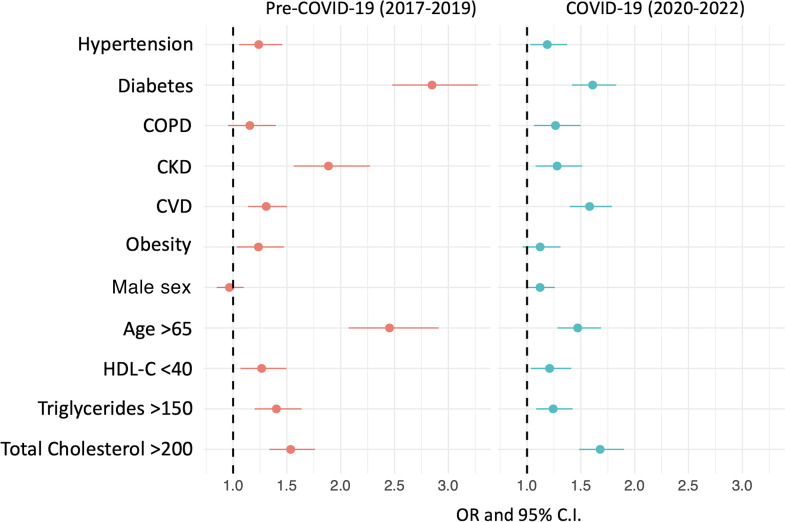
ORs for dyslipidemia with respect to the 3 principal components (i.e., abnormal lipid laboratory results, shown in italics) and to the composite outcome for individuals with different demographic factors (sex and age) and comorbidities (namely hypertension, diabetes, COPD, CKD, obesity) stratified according to the pre–COVID-19 and COVID-19 periods. See also Supplemental Tables 1 and 2.

In order to further highlight the role of the COVID-19 pandemic on the incidence of the key components of the definition of dyslipidemia and among individuals of different ages, sex, and comorbidities, we stratified the results obtained according to the pre–COVID-19 and COVID-19 periods. As shown in Figure 4, this analysis indicated that during the COVID-19 period, only the OR for total cholesterol levels above 200 mg/dL did, in fact, increase, whereas the risk of the composite outcome was reduced in patients with diabetes or CKD and in individuals older than 65 years of age, as shown in Supplemental Tables 1 and 2; supplemental material available online with this article; https://doi.org/10.1172/JCI183777DS1

## Discussion

The main finding of our study is the observation that the increased risk of dyslipidemia during the COVID-19 pandemic involved not only patients who had SARS-CoV-2 infection but the whole population.

To our knowledge, our research is the first to be conducted on a large sample of the general population rather than relying on hospital or outpatient clinic databases. In Italy, every citizen has a primary care physician (family doctor), who provides care even in the absence of specific health issues. Consequently, the database we used encompassed not only patients with diagnosed conditions but also individuals without known illnesses (or those unaware of any). This situation is particularly relevant to COVID-19, as the reduction in symptoms and the lack of thorough infection tracking have led many people to contract the virus without realizing it or reporting it to health authorities.

While we believe that a history of COVID-19 infection influences the risk of dyslipidemia, we also recognize that the reported positivity rate in our sample is significantly underestimated compared with the prevalence observed in Italy (approximately 40%, varying by region). For instance, in Campania (the region in which Naples is located), there have been over 2.4 million COVID-19 cases during the 3 years of the pandemic among a total population of 5.5–6 million. Given the significant underreporting of COVID-19 positivity in our dataset, we opted not to include this information in our model, as doing so could have introduced bias.

Having access to data on the population in the years before and during the COVID-19 pandemic constitutes a major strength of our study. In this manner, we successfully addressed the challenge of unawareness of the disease, which has been a significant limitation in prior studies because it is impossible to guarantee that the control groups are genuinely free of infection.

The analyses conducted by Xu and collaborators (1) had shown that patients with COVID-19 face a heightened risk of dyslipidemia and are more likely to be on lipid-lowering medications, which implies a greater susceptibility to developing cardiometabolic disorders. However, these assessments did not elucidate the potential effect of this augmented risk on the overall burden of cardiometabolic diseases or its implications for health care systems and costs. Our observation that the risks of incident composite dyslipidemia outcomes were evident in subgroups on the basis of age, obesity, CVD, CKD, COPD, diabetes, and hypertension is in agreement with the results of Xu et al. (1), with the only difference being that in their study, males had a greater risk of developing dyslipidemia, while we failed to confirm this finding, most likely on account of the different female-to-male ratios between the 2 studies.

Our study is not exempt from limitations. First, we recognize that our findings do not permit any conjecture regarding the pathophysiological mechanisms contributing to the heightened incidence of dyslipidemia during the COVID-19 period. These mechanisms could encompass direct effects of SARS-CoV-2 infection, as well as indirect influences such as stress, alterations in diet and physical activity, modifications in cardiovascular prevention strategies, and limited access to health care during the pandemic. Sorokin and colleagues hypothesized that changes in the composition and/or quantity of HDL that occur with COVID-19 could reduce the antioxidative and antiinflammatory effects of HDL and eventually lead to pulmonary inflammation (11). Besides, lipoproteins containing oxidized phospholipids and fatty acids may contribute to virus-related organ injury by excessively activating innate immune scavenger receptors; restoring lipoprotein function using ApoA-I elevating agents or inhibiting these scavenger receptors with neutralizing antibodies could, therefore, be beneficial in the treatment of COVID-19 (12). Second, the pandemic itself, but not necessarily the infection, might have raised plasma lipid levels via increased stress as a consequence of dramatic changes in health strategies, lifestyle, and economic status (13–21). The pandemic crisis has caused great unrest in society and unprecedented changes in lifestyle, work, and social interactions (22). The enforcement of measures like social distancing and the shutdown of gatherings and interaction spaces such as parks, schools, cafes, and similar venues have led to nontrivial social effects (23, 24). Third, although defining the precise molecular mechanisms is beyond the scope of this epidemiologic study, we believe that the sharp increase in the risk of developing dyslipidemia recorded in patients with diabetes during the COVID-19 pandemic deserves further investigation. Finally, weaknesses also include the use of a cohort that was not population based, the potential misclassification of incident versus prevalent cases, and a possible selection bias due to exclusion of large numbers of people.

## Methods

*Sex as a biological variable*. Both males and females were included in this study.

### Study design and participants

The database included participants above the age of 18 years. Individuals with any history of abnormal lipid laboratory results or lipid-lowering medication prescriptions on January 1, 2017 were excluded. The COVID-19 diagnosis was established on the basis of a documented positive PCR test, as we described previously (8, 25–27), or following a previous hospital admission with a confirmed COVID-19 diagnosis.

The COMEGEN database gathers diagnoses following the International Classification of Diseases 10 (ICD-X) (8). Pharmaceutical prescriptions are documented in the COMEGEN database, which includes details such as the date, brand name, and active ingredients, along with the quantities and methods of administration. Additionally, the database contains information on vital signs, weight, height, BMI, chronic conditions, medical visits, hospitalizations, emergency department visits, medication dispensations, testing, and vaccinations (including for COVID-19). These comprehensive data enable real-time tracking of patient management concerning processes and outcomes, drug usage, diagnostic evaluations, and the complexity and comorbidities of the patient population (9). Evaluation of person-time is essential for calculating incidence rates, and the COMEGEN database facilitates a precise assessment in this regard by recording when each individual was entered into the database and began contributing data to the cohort, as well as documenting dates of death, end of follow-up, and end of observation. The COMEGEN database provided demographic and clinical data, along with laboratory measurements and medication information.

### Study outcomes

We specified the composite of any dyslipidemia outcome as the first occurrence of any of the predefined dyslipidemia outcomes (prescription of lipid-lowering medications and/or abnormal lipid laboratory results). Furthermore, we carried out subgroup analyses consisting of age (≤65 years or >65 years), sex (male or female), hypertension, diabetes, obesity (≥30 kg/m^2^), smoking, CVD, and CKD.

### Study variables

Predefined covariates included a prescription record of antidyslipidemic agents, demographic characteristics, clinical characteristics, and comorbidities (28–31). Specifically, demographic characteristics included age, sex, BMI, and smoking status (never, former, or current). Clinical characteristics included systolic/diastolic BP readings and estimated glomerular filtration rate (eGFR). Comorbidities included CVD, CKD, COPD, diabetes, and hypertension.

CVD was defined as heart failure, coronary heart disease, myocardial infarction, or stroke. CKD was defined according to the eGFR, which was calculated according to the 2021 race- and ethnicity-free CKD Epidemiology Collaboration creatinine equation (32, 33), and participants were classified into 4 CKD risk levels: low, moderate, high, and very high (34); moderate- or higher-risk levels of CKD were considered CKD in the current analysis (35–37). Diabetes was defined as the presence of type 2 diabetes, which was established by either diagnosed diabetes or antidiabetes therapy and also by a glycated hemoglobin level of at least 6.5% in the absence of a diagnosis (undiagnosed diabetes) (38, 39). Hypertension was defined as a record of systolic BP above 140 mmHg and/or diastolic BP above 90 mmHg, or a previous diagnosis of hypertension as defined by the ICD-10 (10th revision of the International Classification of Diseases; code I-10), or a prescription record of antihypertensive medications for more than 30 days.

### Statistics

Categorical variables are reported as absolute frequencies and percentages, whereas continuous variables are reported as means and SD. The Shapiro-Wilk normality test was used to assess the normal distribution of the data. Differences between pre–COVID-19 (i.e., in the 3-year period of 2017–2019) and COVID-19 (i.e., in the 3-year period of 2020–2022) groups in baseline characteristics were tested by Pearson χ^2^ or Fisher’s exact tests for categorical variables and by Welch’s *t* test for continuous variables.

To characterize the individuals affected by dyslipidemia, the following criteria were considered (note that the patients who already had any of these conditions on January 1, 2017 and January 1, 2020, respectively, in the pre–COVID-19 and COVID-19 groups were excluded): total cholesterol above 200 mg/dL, HDL cholesterol below 40 mg/dL, triglyceride levels above 150 mg/dL, and on lipid-lowering therapy.

Given the difference between the pre–COVID-19 and COVID-19 groups in terms of baseline characteristics, propensity scores were calculated and used to match the 2 groups with respect to the baseline covariates (i.e., age, sex, BMI, smoking habit, creatinine, systolic BP, diastolic BP, total cholesterol, HDL cholesterol, and triglycerides). The propensity score is the conditional probability of having the treatment given a vector of measured covariates (40). Propensity score matching was performed as we previously described (40), according to the step-by-step process outlined below.

#### Selection of covariates.

The baseline covariates considered for the propensity score calculation were selected on the basis of their clinical relevance and potential to influence the outcomes of interest. The selected covariates included: age, sex, BMI, smoking habit, creatinine levels, systolic BP, diastolic BP, total cholesterol, HDL cholesterol, and triglycerides.

#### Propensity score estimation.

We used a multivariable logistic regression model to estimate the propensity score, which represents the conditional probability of being in the COVID-19 group given the measured covariates. This logistic regression model included the selected covariates as predictors and the group assignment (pre–COVID-19 vs. COVID-19) as the outcome. The model was fitted using the MatchIt R package with a probit link function.

#### Matching algorithm.

Once the propensity scores were estimated, we performed the matching using a nearest-neighbor approach (40). This method paired each individual in the COVID-19 group with an individual from the pre–COVID-19 group who had the closest propensity score, within a defined tolerance (caliper). The key parameters for the matching process were (a) the caliper width, set at 0.2 of the SD of the logit of the propensity score, which restricts the maximum allowable difference in propensity scores between matched pairs to minimize bias and (b) the matching ratio, in this case, a 1:1 matching ratio, meaning each individual in the COVID-19 group was matched to 1 individual from the pre–COVID-19 group.

#### Final matched dataset.

After the matching process, the final matched dataset was obtained using the MatchIt R package. This dataset included only the pairs of individuals whose propensity scores fell within the defined caliper. The balance of covariates between the 2 groups was assessed by calculating the standardized mean differences (SMDs) for each covariate, along with their 95% CIs, both before and after matching. SMD values below 0.1 were considered indicative of a good balance between the 2 groups.

#### Outcome of interest.

It is important to note that the propensity score model was designed to balance baseline covariates across the groups rather than to predict the outcome of interest

#### Diagnostics and assessment of matching quality.

To verify the effectiveness of the matching, we plotted the distribution of propensity scores in both groups before and after matching. Additionally, we compared the baseline covariates between the groups using SMDs. The matching process was deemed successful if the SMD for each covariate was statistically significant at 5% (i.e., CIs at a 95% of confidence degree include the zero value).

A multivariable logistic regression model was performed to estimate the effect of the potential group risk factor of dyslipidemia. In particular, ORs estimated by logistic regression measured the probability of developing dyslipidemia in the COVID-19 group on the probability of developing dyslipidemia in the pre–COVID-19. The OR estimates were adjusted for demographic and clinical factors. The same approach was used to estimate the effect of the 3 principal components (i.e., the 3 abnormal lipid laboratory results except the lipid-lowering therapy because it had a strong association with these 3 factors) on developing dyslipidemia within the pre–COVID-19 and COVID-19 cohorts. The OR estimates were adjusted for demographic and clinical factors.

Statistical analyses were performed using R (version 4.4.1, R Foundation for Statistical Computing), SPSS (Statistical Product and Service Solutions, version 29), or GraphPad Prism (version 10.1.2, Dotmatics). A *P* value of less than 0.05 was considered significant.

#### Study approval.

This study adhered to the Strengthening the Reporting of Observational Studies in Epidemiology (STROBE) guidelines. The Ethics Board at ASL Napoli 1 Centro (Naples, Italy) reviewed and approved the study, granting a waiver of informed consent due to the anonymized nature of the data collection (no. 257/22-2023).

#### Data availability.

Data supporting the figures are provided in the Supporting Data Values file. Raw data are available at the following link: https://doi.org/10.6084/m9.figshare.26808625 Other data are available upon reasonable request to the corresponding author, subject to institutional review and approval.

## Author contributions

G Santulli and BT were responsible for designing research studies, acquiring data, analyzing data, and writing the manuscript. VT, RI, SSJ, MF, G Signoriello, MVM, ML, FR, PM, and FV were responsible for data collection and analyses. PG, GE, RP, CM, and GP contributed to data curation and critical revision of the manuscript. All authors agreed on the content of the manuscript, reviewed drafts, and approved the final version. All authors had full responsibility for the decision to submit for publication. The order of co–first authors’ names was determined by drawing lots.

## Supplementary Material

Supplemental data

ICMJE disclosure forms

Supporting data values

## Figures and Tables

**Table 2:**
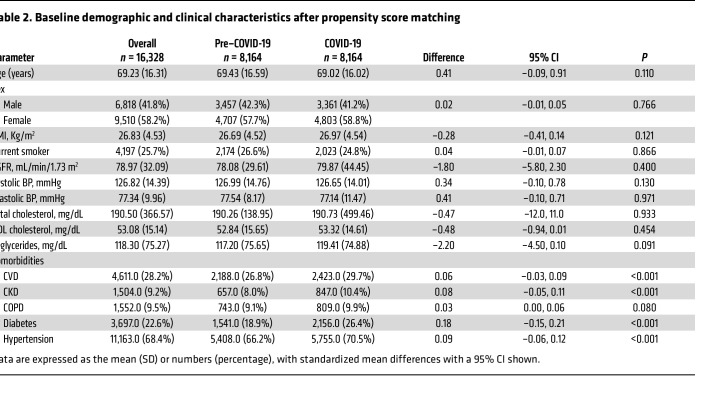
Baseline demographic and clinical characteristics after propensity score matching

**Table 1:**
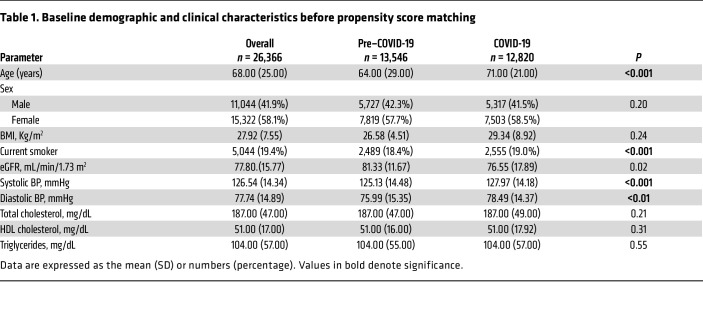
Baseline demographic and clinical characteristics before propensity score matching

## Version 1 09/12/2024

In-Press Preview

## Version 2 11/01/2024

Electronic publication
