# Supplementary material for: A six-year study in a real-world population reveals an increased incidence of dyslipidemia during COVID-19
Source: J Clin Invest. 2024 Sep 12;134(21):e183777. doi: 10.1172/JCI183777 (PMC11527440; doi:10.1172/JCI183777)
Supplement: Supplemental data [file jci-134-183777-s088.pdf]

SUPPLEMENTARY MATERIAL

A six-year study in a real-world population reveals an increased incidence of dyslipidemia during COVID-19

Valentina Trimarco,[1,#] Raffaele Izzo,[2,#] Stanislovas S. Jankauskas,[3,#] Mario Fordellone,[4] Giuseppe Signoriello,[5] Maria Virginia Manzi,[2] Maria Lembo,[2] Paola Gallo,[1] Giovanni Esposito,[2] Roberto Piccinocchi,[6] Francesco Rozza,[2] Carmine Morisco,[2,7,8] Pasquale Mone,[3,9,10] Gaetano Piccinocchi,[11] Fahimeh Varzideh,[3] Bruno Trimarco,[2,8] and Gaetano Santulli[2,3,8,12,*]

[1]Department of Neuroscience, Reproductive Sciences, and Dentistry and [2]Department of Advanced Biomedical Sciences, "Federico II" University, Naples, Italy.
[3]Department of Molecular Pharmacology, Fleischer Institute for Diabetes and Metabolism (FIDAM), Einstein Institute for Aging Research, Albert Einstein College of Medicine, New York, New York, USA.
[4]Department of Mental, Physical Health and Preventive Medicine and [5]Department of Experimental Medicine, University of Campania "Luigi Vanvitelli," Naples, Italy.
[6]"Luigi Vanvitelli" Hospital, Naples, Italy.
[7]Italian Society for Cardiovascular Prevention (SIPREC), Rome, Italy.
[8]International Translational Research and Medical Education (ITME), Academic Research Unit, Naples, Italy.
[9]Department of Medicine and Health Sciences "Vincenzo Tiberio," Molise University, Campobasso, Italy.
[10]Casa di Cura Montevergine, Mercogliano, Avellino, Italy.
[11]COMEGEN Primary Care Physicians Cooperative, Italian Society of General Medicine (SIMG), Naples, Italy.
[12]Department of Medicine, Division of Cardiology, Einstein–Mount Sinai Diabetes Research Center (ES-DRC), Wilf Family Cardiovascular Research Institute, Albert Einstein College of Medicine, New York, New York, USA.

***Correspondence to:**
Prof. Gaetano Santulli, MD, PhD, FAHA
1300 Morris Park Avenue
10461 New York City, NY
Email address: gsantulli001@gmail.com
Phone: +17184303370

#: Share the first Authorship

Parameter	OR	95% CI	p-value
**Total cholesterol >200 mg/dL**	1.535	1.337, 1.761	<0.001
**HDL cholesterol <40 mg/dL**	1.266	1.068, 1.496	0.006
**Triglycerides >150 mg/dL**	1.402	1.200, 1.636	<0.001
**Age >65 years**	2.453	2.074, 2.910	<0.001
**Male gender**	0.966	0.848, 1.100	0.60
**Obesity**	1.236	1.034, 1.472	0.019
**CVD**	1.308	1.139, 1.501	<0.001
**CKD**	1.886	1.563, 2.271	<0.001
**COPD**	1.155	0.952, 1.397	0.14
**Diabetes**	2.848	2.478, 3.273	<0.001
**Hypertension**	1.238	1.055, 1.457	0.009

Supplementary Table 1

Odds ratios (OR) and confidence intervals (CI) calculated during the pre-COVID-19 period.

Parameter	OR	95% CI	p-value
**Total cholesterol >200 mg/dL**	1.679	1.483, 1.901	<0.001
**HDL cholesterol <40 mg/dL**	1.210	1.036, 1.410	0.016
**Triglycerides >150 mg/dL**	1.243	1.084, 1.423	0.002
**Age >65 years**	1.471	1.282, 1.688	<0.001
**Male gender**	1.121	0.999, 1.258	0.052
**Obesity**	1.122	0.959, 1.310	0.15
**CVD**	1.581	1.398, 1.788	<0.001
**CKD**	1.279	1.080, 1.511	0.004
**COPD**	1.265	1.066, 1.497	0.007
**Diabetes**	1.610	1.417, 1.827	<0.001
**Hypertension**	1.188	1.030, 1.372	0.019

Supplementary Table 2

Odds ratios (OR) and confidence intervals (CI) calculated during the COVID-19 period.
